# A novel naproxen derivative capable of displaying anti-cancer and anti-migratory properties against human breast cancer cells

**DOI:** 10.1186/1471-2407-14-567

**Published:** 2014-08-07

**Authors:** Jolly Deb, Joydeb Majumder, Sankar Bhattacharyya, Siddhartha Sankar Jana

**Affiliations:** Department of Biological Chemistry, Indian Association for the Cultivation of Science, 2A & 2B Raja S. C. Mullick Road, 700032 Kolkata, India; Department of Organic Chemistry, Indian Association for the Cultivation of Science, 2A & 2B Raja S. C. Mullick Road, 700032 Kolkata, India; Department of Zoology, Sidho Kanho Birsha University, Purulia, India

**Keywords:** MCF-7, MDA-MB-231, MTT assay, Apoptosis, Inflammation, Metastasis

## Abstract

**Background:**

Increasingly, the role of chronic inflammation and its mediators in tumor generation and progression is gaining importance in the field of cancer research. In this context, candidature of non steroidal anti-inflammatory drugs (NSAIDs) as potential anti-tumor therapeutic agent is being evaluated globally. In the present study we have evaluated the anti-cancer effect of a series of newly synthesized naproxen derivatives on human breast cancer cell lines.

**Methods:**

MCF-7 (poorly invasive) and MDA-MB-231 (highly invasive) cells were treated with different concentrations of naproxen sodium and its derivatives *in vitro*, and the underlying mechanism of action was monitored by employing studies related to induction of apoptosis, activation of caspases, cell-cycle progression, synthesis of PGE_2_ and cellular migration.

**Results:**

After a preliminary screening using MCF-7 and MDA-MB-231 cells, it was evident that naproxen derivative **4** has a better killing property compared to its parent compound naproxen sodium (NS). On further investigation, it was apparent that the observed growth inhibitory activity on MDA-MB-231 cells after treatment with **4**, was not due to cell cycle arrest but due to an early induction of apoptosis and subsequent induction of caspases 3 and 9. Derivative **4** could also inhibit COX activity in MDA-MB-231 cells as evidenced by reduction in prostaglandin E2 secretion. Moreover, **4** was capable of delaying the overall migration rate of MDA-MB-231 cells *in vitro*.

**Conclusion:**

In this study we report that a naproxen-derivative (**4**) has powerful anti-inflammatory and anti-tumor properties as it induces appreciable amount of apoptosis in breast cancer cell line, and can also delay migration of cancer cells (MDA-MB-231) which would in turn delay cancer cell invasion and formation of secondary tumours in primary breast cancer patients. Thus, we propose that **4** is worthy of further investigation due to its potential as a therapeutic agent in anti-tumor treatment regimen.

## Background

The functional relationship between inflammation and cancer was first brought into picture in 1863, when Rudolf Virchow hypothesized that cancer originates at the site of chronic inflammation [1]. Since then a vast number of studies have emphasized on the role of chronic inflammation in tumorogenesis [2] and potential use of non-steroidal anti-inflammatory drugs (NSAID) as anti-cancer agents [3–5]. Although gastrointestinal bleeding and increased cardiovascular (CV) problems are associated with most of the known NSAIDs, naproxen is known for its fewer CV effects with a possible cardioprotective role in human [6]. In a phase II clinical trial, naproxen was found to be safe and effective in treating progressive prostate cancer with early recurrent disease [7] and a recent study using a murine model of induced colon cancer, has also indicated the anti-cancer properties of this drug [8].

The molecular mechanisms, by which NSAIDs impart their chemopreventive effects, are a matter of intense debate till date. The most accepted hypothesis has focused on their property to reduce the levels of prostaglandins by cyclooxygenase (COX-1 and -2) inhibition [9]. Over expression of COX-2 [10] along with increased levels of prostaglandin E_2_ (PGE_2_) in breast cancer patients have been reported [11]. It is of note that PGE_2_, a known COX-2-derived prostaglandin, plays a significant role in progression and metastasis of cancer cells by modulating local tumor microenvironment [12]. Hence, molecules with an enhanced capacity to reduce PGE_2_ in cancer cells are of great interest.

**Figure 1:**
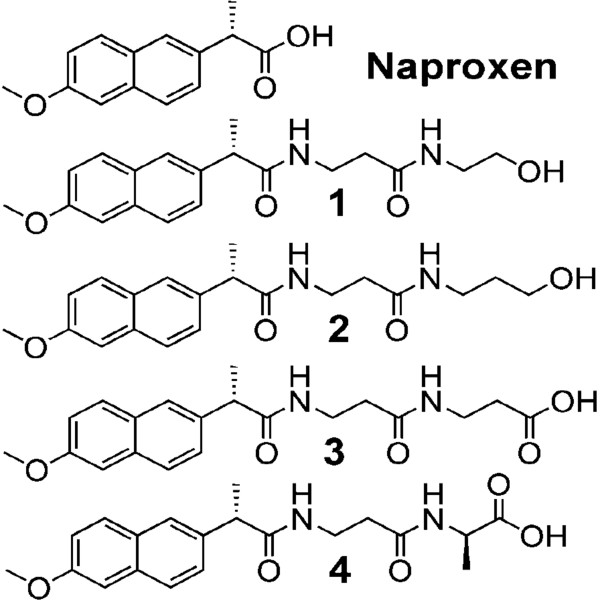
**Naproxen and its derivatives (1–4).**

To demonstrate the above hypothesis we chose to work with four naproxen-derivatives [13] **1**–**4** (Figure 1). In the present study we report that naproxen derivative **4** can be employed as an anti-cancer agent due to its enhanced cytotoxic activity against human breast cancer cell lines and also address the underlying mechanism of action by employing studies related to induction of apoptosis, activation of caspases, cell-cycle progression, synthesis of PGE_2_ and cellular migration.

## Methods

### Synthesis of naproxen derivatives (1–4)

All the naproxen derivatives were synthesized according to the methods described in our previously published literature [13].

### Cell culture

The human breast cancer cell line MCF-7 and the mouse macrophage RAW 264.7 cells were cultured in high glucose Dulbecco's minimal essential medium (DMEM) supplemented with 10% FBS (Life Technologies) and 1% L-glutamine-penicillin-streptomycin (Life Technologies) and Insulin (in case of MCF-7), and maintained in a humidified incubator at 37°C and 5% CO_2._ MDA-MB-231, a rather aggressive form of human breast cancer cell line, was grown in Leibovitz's L-15 medium in presence of 10% fetal bovine serum and antibiotics, penicillin and streptomycin and required a 37°C incubator with 100% air environment for optimum growth. They grew as a monolayer in the culture plate and were regularly passaged at ~80% confluency.

Cell lines were purchased from ATCC (American Type Culture Collection, Manassas, VA, USA) and maintained in our cell culture facility.

### MTT assay

Briefly, 10^4^ cells/well were seeded in a 96-well plate and left to adhere overnight. Compounds were prepared as a stock (50 mM) in DMSO-media, prior to addition to the cells. The culture medium was replaced with medium containing the compounds at various concentrations (1 mM, 3 mM, 5 mM, 10 mM) and incubated for another 24 h following addition of 100 μl of 3-(4,5-Dimethylthiazol-2-yl)-2,5-diphenyltetrazolium bromide (MTT; 1 mg/ml) stain per well, incubated for 3 h at 37°C and subjected to DMSO (150 μl/well). Incubation was carried out further for another 10 min at 37°C, and the absorbance was recorded at 570 nm using a plate reader (VARIOSKAN, ThermoFisher).

### Apoptosis assay

Following treatment of cells (1.5 × 10^6^ MDA-MB-231 cells/well in a 12-well plate) with naproxen sodium or **4** for two different time points, apoptosis was determined by “Apo-TRACE Apoptotic Cell Staining Kit” (# CS1110; Sigma), as per manufacturer’s instruction. Control cells were treated with DMSO-media. In principle, viable cells are Apo-TRACE^-^/PI^-^, dead or necrotic cells are Apo-TRACE^-^/PI^+^, early apoptotic cells are Apo-TRACE^+^/PI^-^, and late apoptotic cells are Apo-TRACE^+^/PI^+^.

### Caspase assay

To detect the levels of caspases-3, -8 and -9 in cell lysates from treated and untreated cells (1.5 × 10^6^ MDA-MB-231 cells/well in a 6-well plate), Caspase Colorimetric Protease Assay Sampler Kit (# KHZ1001; Life Technologies) was used as per manufacturer’s protocol. In principle, active caspase 3, 8 or 9 cleaves the individual fluorogenic substrate to release the fluorochrome, which can be detected using a microtiter plate reader at 405 nm.

### Cell cycle study

**Figure 2:**
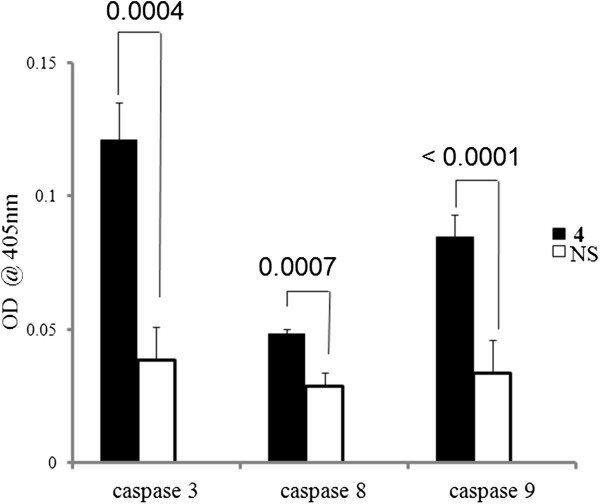
**Derivative 4 induces caspase 3/9 activation.** MDA-MB-231 cells were left untreated or treated with NS and **4** for 6 h, and cell lysates were prepared. The lysates were then incubated with substrates of caspase 8, 9 and 3, and OD value at 405 nm was measured using a standard plate reader. Treatment with **4** resulted in a significant increase in the activation of caspase -9 and -3 compared to its parent compound NS. Each point on the graph represents Mean ± SD; p values of *t*-test are indicated on top of horizontal bars. n = 3.

In order to determine the cell cycle phase distribution of nuclear DNA, cells (1.5 × 10^6^ MDA-MB-231 cells/well in a 6-well plate) were harvested and fixed in 100% chilled methanol and permeabilized. Cells were treated with RNase (10 μl from 2 mg/ml stock) and nuclear DNA was labeled with PI (125 μg/ml). Cell-cycle phase distribution was determined on a FACSCalibur using CellQuest software [14]. For each sample, 10,000 events were acquired for analysis after excluding cell doublets and clumps by gating. Histogram display of FL2-A (x axis, PI-fluorescence) versus counts (y axis) has been displayed in Figure 2.

### PGE_2_ synthesis assay

The amount of secreted PGE_2,_ from treated and untreated cell (1.5 × 10^6^ MDA-MB-231 cells/well in a 12-well plate) culture supernatants, was determined using Prostaglandin E2 EIA kit (Cayman Chemicals, Ann Arbor, MI). The conditioned medium was centrifuged to remove any cells and stored immediately at -70°C until assay. The assay was done according to the manufacturer’s protocol.

### Cell migration assay

MDA-MB-231 cells were grown to confluency in a 9 cm^2^ culture plate. A 200 μl sterile pipette tip was used to introduce a scratch followed by treatment with different concentrations (1 mM and 3 mM) of naproxen sodium, **4** or DMSO. Still and/or video images were captured during or after 22 h to monitor wound closure rate to measure migration speed and polarity [15].

### Statistical analysis

IC_50_ was calculated by nonlinear regression analysis using OriginPro. The mean and SD values were computed using Microsoft Excel, and GraphPad software was used to calculate p values.

## Results and discussion

### Enhanced cytotoxic killing is evident by naproxen-derivatives against MCF-7 and MDA-MB-231 cells

**Figure 3:**
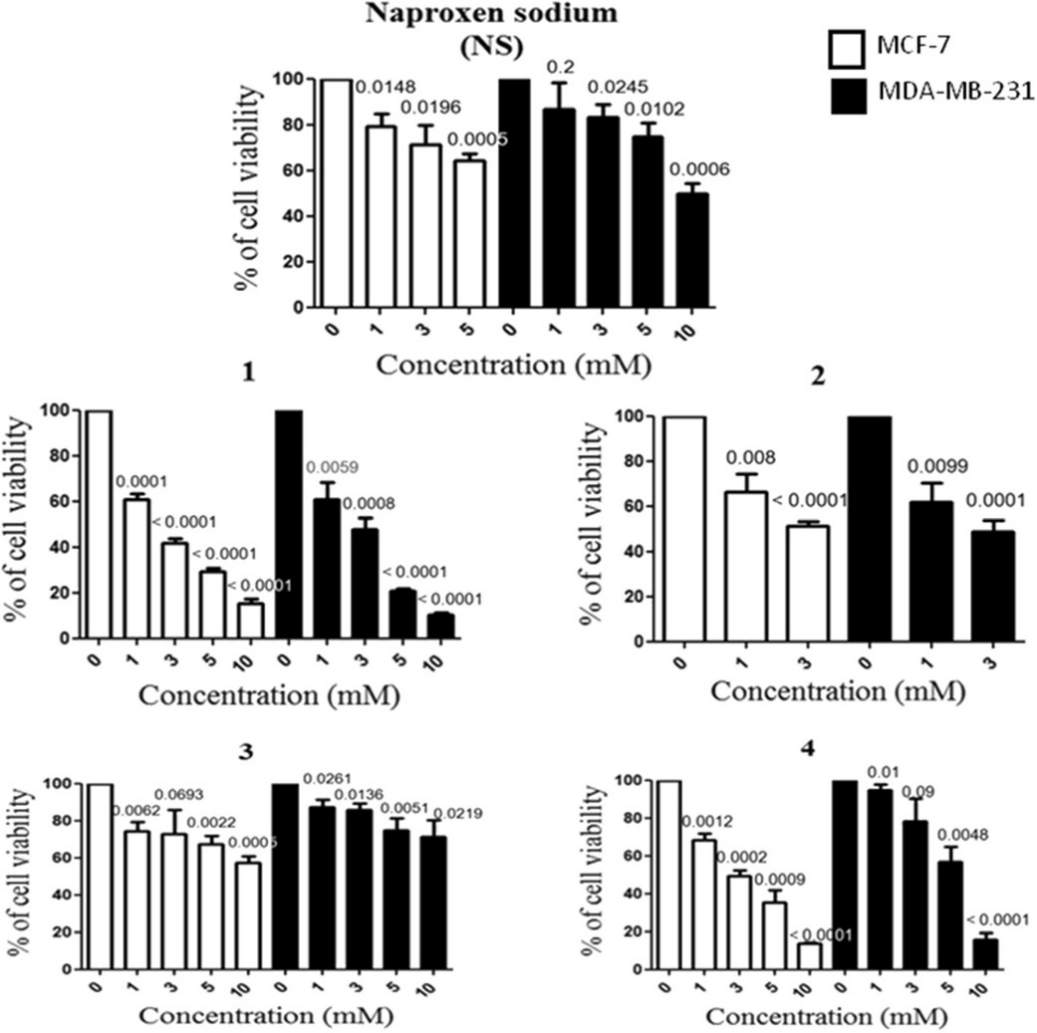
**Naproxen-derivatives induce cytotoxic killing in two breast cancer cell lines.** MCF-7 and MDA-MB-231 cells were treated with different concentrations of naproxen sodium (NS) and its derivatives for 24 h followed by addition of MTT. Except **3**, rest of the derivatives showed an enhanced killing effect on both the cell lines, when compared with NS. Each point on the graph represents Mean ± SD. n = 3.

The cytotoxic effect of the synthesized molecules, the naproxen-derivatives (Figure 1), was first examined in a preliminary screening using two human breast cancer cell lines, MCF-7 and MDA-MB-231 cells (Figure 3) and the overall findings have been summarized in Table 1. Based on their IC_50_ values, except **3,** rest of the derivatives were found to have a better killing property compared to their parent compound naproxen sodium (NS). It is of note that although **1** and **2** could exhibit better killing properties (IC_50_ ~ 2 mM and ~3 mM, respectively) against the cancer cell lines, they also proved to be cytotoxic against the mouse macrophage cell line RAW 264.7 [13]. Because macrophages serve as the first line of defense during tumor establishment [16], a drug molecule with little to no toxic effect against macrophages would be advantageous. Keeping these in mind, we conclude that **4** has a better cytotoxic effect against the two cancer cell lines employed in the study with minimal adverse effect on RAW 264.7 cell.

**Table 1:** **In vitro cytotoxicity of naproxen sodium and its derivatives**

24-h IC _50_, mM		
Compound	MCF-7	MDA-MB-231
	ER ^+^, PR ^+^	ER ^-^, PR ^-^, HER ^2^/Neu ^-^
Naproxen sodium (NS)	>5	~10
1	~1.8	~1.6
2	~3	~3
3	>10	>10
4	~2.7	~5.9

### Treatment with 4 induces early apoptosis in MDA-MB-231 cells

**Figure 4:**
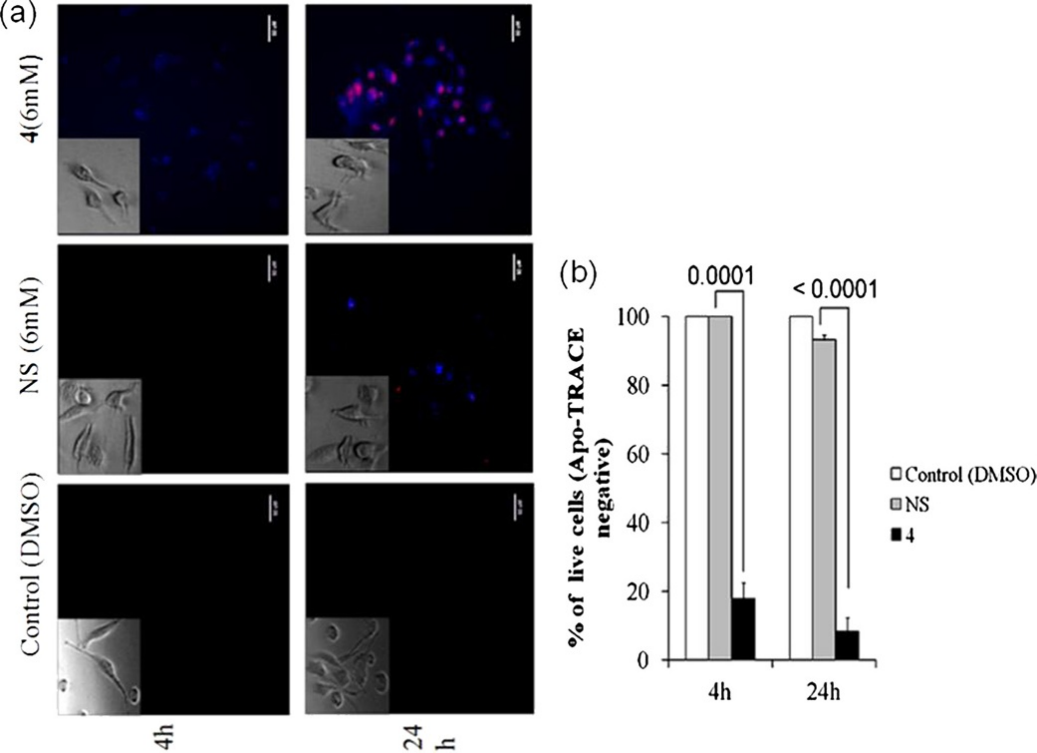
**Derivative 4 can induce apoptosis in MDA-MB-231 cells.** MDA-MB-231 cells were incubated with or without cytotoxic compounds for 4 h and 24 h, and were stained with the Apo-TRACE cell staining kit followed by fluorescence imaging studies. **(a)** Cells treated with **4** lead to an early induction of apoptosis than NS. (Inset showing bright field image of control and drug treated MDA-MB-231 cells indicating morphological changes in cell structure upon drug treatment.) At least 3 microscopic fields were examined for staining and representative images are shown here. **(b)** Bar graph representing percentage of live cells. Each point on the graph represents Mean ± SD. n = 3.

Because MDA-MB-231 is a highly aggressive cell line compared to MCF-7, we sought to study our compound of interest in depth using the former one. In order to understand whether the observed cell killing was due to apoptosis or necrosis, we looked for Apo-Trace positive cells in drug-treated samples. We could detect early apoptotic cells within the first 4 h of **4** (6 mM) treatment and late apoptotic cells at a later time point (24 h). Cells treated with NS at the same concentration could not undergo apoptosis by 4 h and very few apoptotic cells were evident at 24 h time point (Figure 4).

### Naproxen derivative 4 induces caspase-3/9 mediated apoptosis in MDA-MB-231 cells

Many researchers have commented on the crucial role of caspase-cascade system in apoptosis, and the role of caspase-8, -9 as executioner and -3 as death protease [17] is well accepted. Treatment with **4** (6 mM) for 6 h resulted in a significant increase in the activation of caspase -9 and -3 compared to its parent compound NS (Figure 2). These results suggest that the **4** mediated observed apoptosis in MDA-MB-231 cells is due to activation of caspases-3 and -9. Hence, our study clearly indicates involvement of the intrinsic pathway [18] of caspase activity in inducing apoptosis in MDA-MB-231 cells.

### Neither NS nor 4 induces cell-cycle arrest in MDA-MB-231 cells

**Figure 5:**
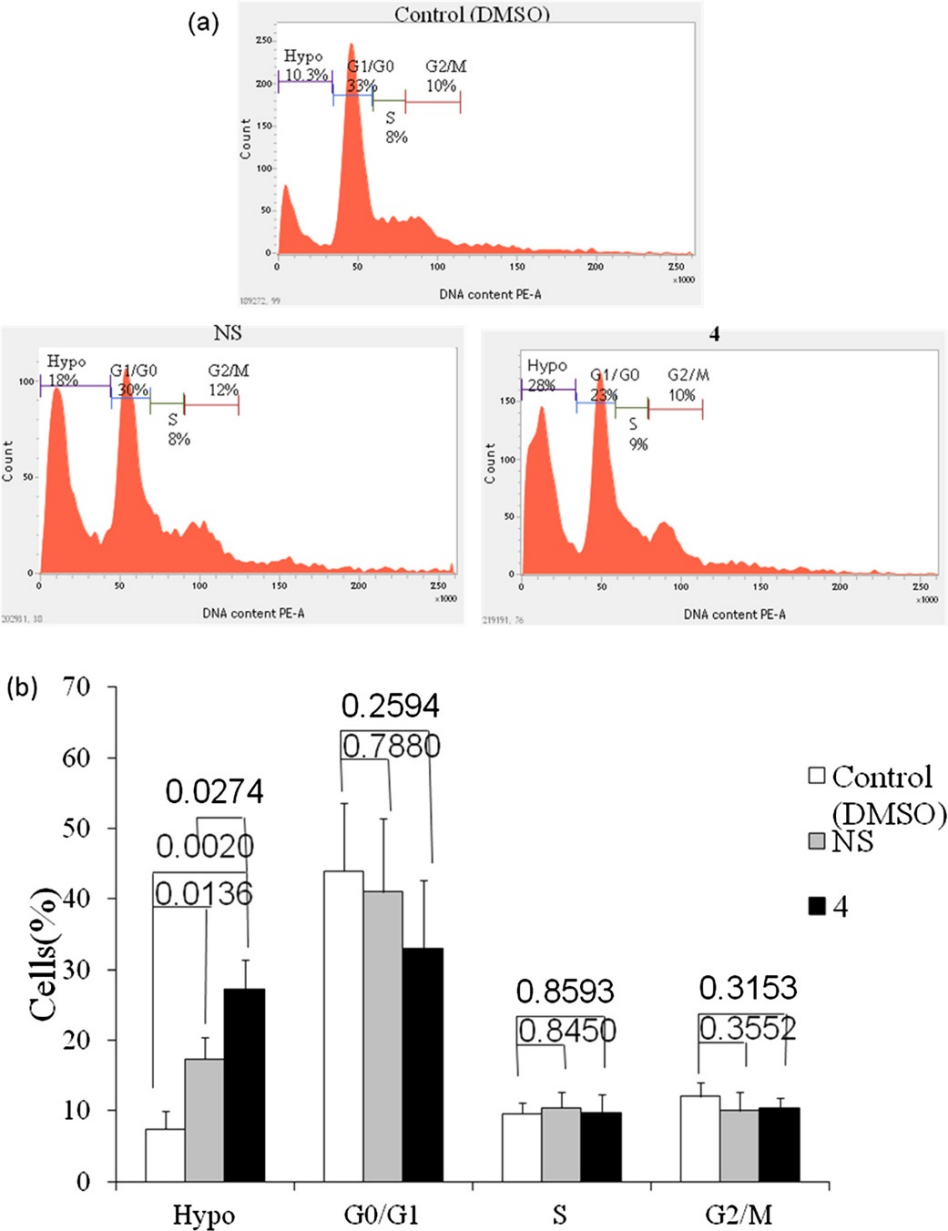
**Cell cycle arrest in MDA-MB-231 cells is not evident upon treatment with 4.** MDA-MB-231 cells were treated with DMSO or cytotoxic compounds for 24 h. Followed by fixation and permeabilization, the cells were stained with PI. **(a)** Cell-cycle phase distribution was determined by flow cytometric analysis. **(b)** No significant cell-cycle arrest in cells treated with either NS or **4** was detected. Each point on the graph represents Mean ± SD, p values of *t*-test are indicated on top of horizontal bars, n = 3.

Growing bodies of evidence suggest that molecules, capable of affecting cell cycle progression in cancer cells, can enhance sensitivity of cancer cells towards anti-cancer drugs [19, 20]. As our study could clearly indicate an increase in apoptotic cell death after treatment with **4**, we carried out cell-cycle analysis to detect any changes in cell-cycle progression (Figure 5). We failed to notice significant cell-cycle arrest in cells treated with NS or **4** for 24 h compared to DMSO treated control cells. However, in drug-treated samples, we could detect a high percentage of cell population residing in the hypoploid state, indicating apoptotic cell population. Hence, both NS and its derivative **4** mediated observed growth inhibitory activity on MDA-MB-231 cells was not due to cell cycle arrest but due to induction of apoptosis.

### Naproxen derivative 4 inhibits PGE_2_ synthesis in MDA-MB-231 cells

**Figure 6:**
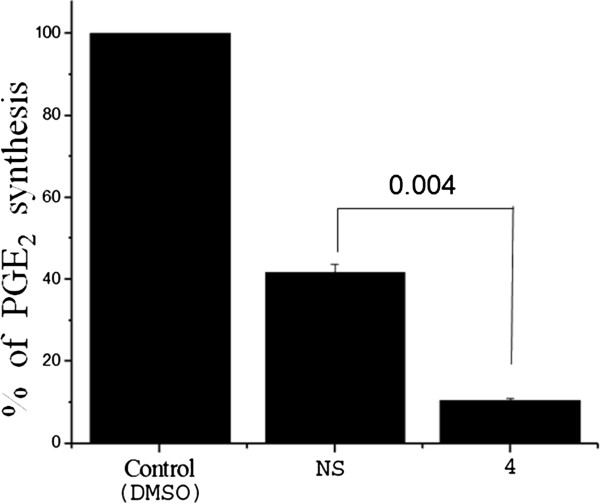
**Derivative 4 inhibits Prostaglandin E2 secretion in MDA-MB-231 cells.** Conditioned medium from 24 h treated or untreated culture wells were collected and PGE_2_ levels were assayed using Prostaglandin E2 EIA kit. At 6 mM concentration, **4** could significantly reduce PGE_2_ secretion in MDA-MB-231 cells compared to NS. Each point on the graph represents Mean ± SD, p values of *t*-test are indicated on top of horizontal bars. n = 3.

A hallmark of breast cancer cells is up-regulated COX-2, which in turn result in an increase in PGE_2_ synthesis [21]. As NSAIDs are known to affect COX-2 activity and reduce COX-2 induced PGE_2_ synthesis, we wanted to determine if **4** could also act in the similar fashion. We checked the level of PGE_2_ in conditioned cell culture medium collected from MDA-MB-231 cells after DMSO or drug-treatment for 24 h. At 6 mM concentration, **4** could significantly reduce PGE_2_ secretion by MDA-MB-231 cells compared to NS (Figure 6), confirming **4** to be a more potent inhibitor of COX-2 mediated PGE_2_ production. The observed phenomenon could be due to inhibition of COX-2, as the parent compound Naproxen is a well known COX inhibitor. This finding might also establish **4** as a promising candidate in reducing inflammation which is known to play significant role in cancer progression [22].

### Treatment with 4 can slow-down cell-migration rate in MDA-MB-231cells

**Figure 7:**
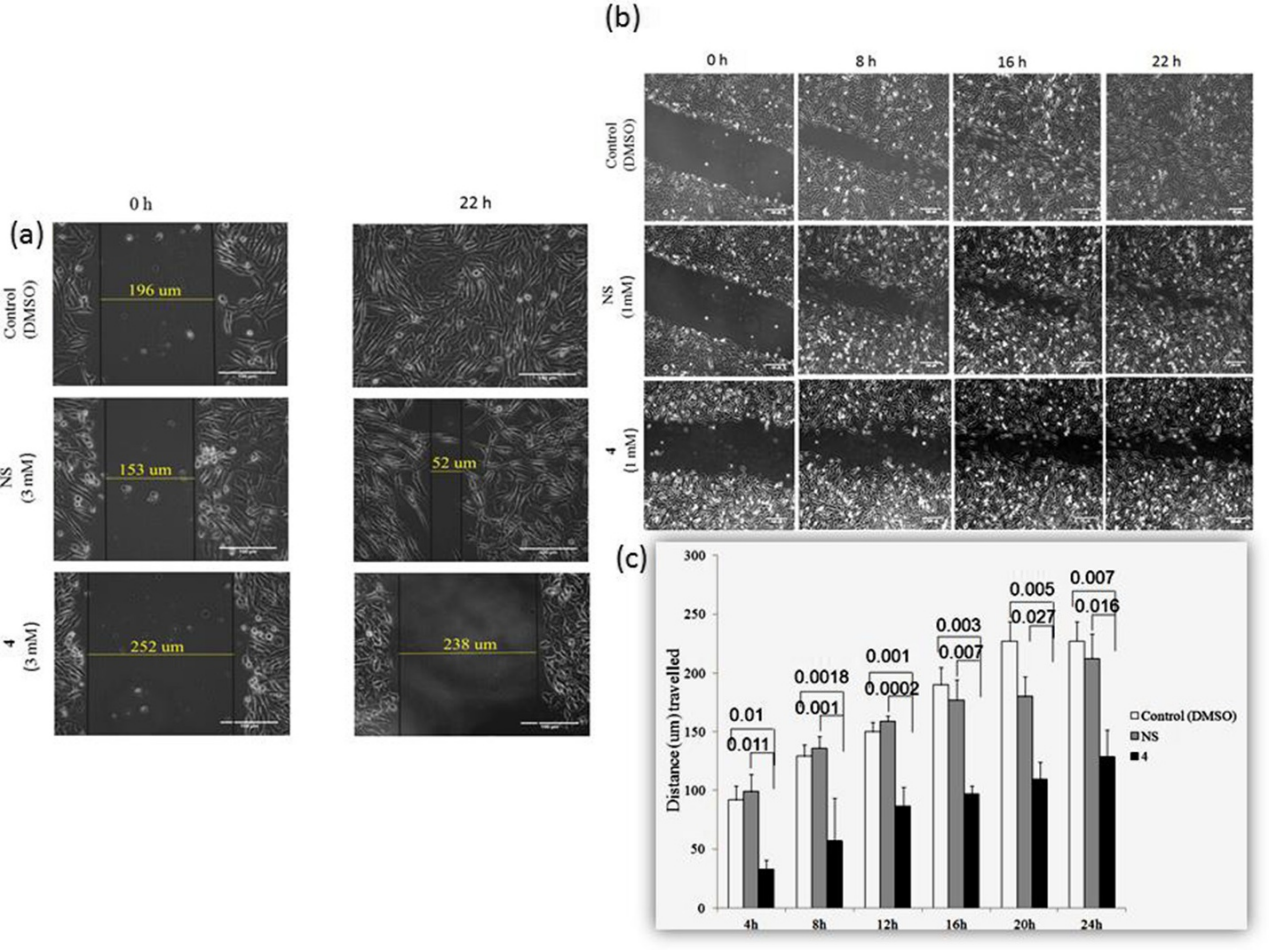
**Treatment with 4 delays migration rate of MDA-MB-231 cells** ***in vitro*** **.** A scratch was introduced in the culture dish and the cells were left treated (3 mM and 1 mM) or untreated for 22 h. Photo micrographic as well as time-lapse video micrographic images were captured to study relative migration capacity of control or drug treated MDA-MB-231 cells. **(a)** At 3 mM concentration, cells treated with NS covered 66% of the gap, whereas, **4**, could cover only 6% of the gap. **(b)** Video micrographic images indicating migration rates of control or treated MDA-MB-231 cells during 22 h. **(c)** Graph indicating distance travelled/h of control or treated MDA-MB-231 cells during 22 h. Each point on the graph represents Mean ± SD, p values of *t*-test are indicated on top of horizontal bars. n = 3.

After evaluating the effect of **4** in the production of PGE_2,_ we wanted to test the influence of **4** on the cancer cell migration as it is now well established that PGE_2_ plays a vital role in the migration of breast cancer cells. Poor prognosis of breast cancer patients are associated with secondary tumor formation, thus, any compound that can intervene with migrational capacity and metastasis of breast cancer cells may have profound impact on improved survivability of such patients. We opt for MDA-MB-231 over MCF-7 due to its highly metastatic and migratory properties compare to the non- metastatic and poorly migrating MCF-7 cells [21]. Cells treated with NS for 22 h at a higher concentration (3 mM) covered 66% of the gap, whereas, **4**, at the same concentration and time point, could cover only 6% of the gap (Figure 7a).

We then opted for a lower dose (1 mM) of drug (NS and **4**) to study dose-dependent migratory inhibition in the same cell line, and captured time-lapse video images of treated/untreated cells for duration of 22 h (Figure 7b). We, then, randomly selected seven time points to calculate the migration speed (Table 2) based on the distance travelled/h (Figure 7c). The cells, treated with NS, migrated at a speed of 8.5 μm/h whereas, treatment with **4** led to a much slower rate (4.5 μm/h) of migration; almost half of the migration speed of NS-treated cells. These results clearly indicate that **4** could successfully delay the migration of MDA-MB-231 cells, most probably by reducing COX-2 derived PGE_2_ synthesis. Since migration is the first step towards invasion and metastasis of cancer cells, **4** might as well interfere with the metastasis process. However, further experimentation using murine cancer-model would be essential to support the claim.

**Table 2:** ***In vitro*** **migration speed of control or treated MDA-MB-231 as determined by scratch wound assay**

Drug Treatment (1 mM)	Migration speed (μm/h)
Control	~12
NS	~8.83
4	~5.3

## Conclusions

Here, we have presented a new peptide based derivative **4** obtained from the NSAID naproxen capable of showing cytotoxic property against two human breast cancer cell lines and, have also provided brief experimental evidence, supported by literature, to address the underlying mechanism of action. In summary our studies suggest that derivative **4** mediated cell-killing is due to an early induction of apoptotic event followed by activation of intrinsic caspase-cascade at a molecular level, while no sign of significant cell-cycle arrest was evident. Furthermore, we have showed that **4** can inhibit PGE_2_ synthesis and delay tumor cell migration (*in vitro*). Since the derivative **4** is capable of forming hydrogel at room temperature [13] it can also be employed as the hydrogel drug-delivery system [23] for sustained release of the anti-cancer agent. However, it is also important to note the difference in the IC_50_ vales of **4** against p53 wild type MCF-7 (~3 mM) and p53 null MDA-MB-231 (~6 mM) cells. The highly skewed killing of MCF-7 cells by this compound might indicate the involvement of p53 mediated apoptotic pathway. One of the major off target effects of **4** may include p53 targets such as bax. Further experimentation is needed to elucidate these potential off target effects of this novel anti-cancer compound. Potential limitation of this drug could be the fact that it may include p53 as one of its targets, thus the dose needed to induce significant killing may be relatively elevated for p53 muted cancer cells compared to p53 wild type cancer cells. Overall these studies imply that peptide based naproxen-derivative **4** can serve as an anti-cancer agent.
